# Diagnostic Tools Applied to Determine Thyroid Lipoadenoma: A Case Report

**DOI:** 10.7759/cureus.83096

**Published:** 2025-04-27

**Authors:** Luo-Wei Chan, Jia-Yu Chen, Jui-Ju Kao, Min-Wei Yu, Hsin-Rou Liang

**Affiliations:** 1 Department of Clinical Education and Training, Kaohsiung Medical University Hospital, Kaohsiung, TWN; 2 Department of Pathology, Kaohsiung Medical University Hospital, Kaohsiung, TWN; 3 Department of Trauma Surgery, Kaohsiung Medical University Hospital, Kaohsiung, TWN; 4 Department of General and Digestive Surgery, Kaohsiung Medical University Hospital, Kaohsiung, TWN

**Keywords:** endocrine neoplasms, thyroid lipoadenoma, thyroid nodule, thyroid screening, thyroid surgeon

## Abstract

We report a case of thyroid lipoadenoma of the thyroid gland, an extremely rare benign lesion containing both adipose tissue and thyroid tissue. A 74-year-old Chinese female underwent a series of examinations for the incidental thyroid nodule. The sonogram showed a well-encapsulated and hyper-isoechoic nodule in the left thyroid gland. The CT revealed a low-attenuation thyroid nodule without evidence of extrathyroid extension. Fine-needle aspiration (FNA) cytology reported atypia of undetermined significance. As none of these studies could confirm the diagnosis and rule out potential malignancy, the patient ultimately received minimally invasive video-assisted thyroidectomy (MIVAT). Final pathology confirmed the diagnosis of thyroid lipoadenoma, consisting of adipose tissue and follicular cells, with adipose tissue comprising approximately 40% to 50% of the lesion. Common diagnostic tools for thyroid nodules offer limited utility in identifying thyroid lipoadenoma, often requiring thyroidectomy for definitive diagnosis and management.

## Introduction

Thyroid lipoadenoma, also referred to as thyroid adenolipoma or thyrolipoma, is a rare benign well-encapsulated neoplasm in the thyroid gland, consisting of both adipose tissue and thyroid tissue. Although adipose tissue can be found in other endocrine glands such as the pancreas and parathyroid gland, its presence in the thyroid gland is exceedingly rare. Only a few case reports have documented patients with thyroid lipoadenoma [1].

Most patients with thyroid lipoadenoma are asymptomatic and euthyroid; however, as the tumor enlarges, they may notice palpable masses in their neck. This mass can potentially compress adjacent organs, causing symptoms such as dyspnea, dysphagia, or, in severe cases, airway obstruction [2]. Fine-needle aspiration (FNA) is a cost-efficient, safe, and simple method for evaluating thyroid lesions. In cases of thyroid lipoadenoma, cytologic reports from FNA may show the presence of both adipocytes and follicular cells [3]. A CT may reveal a low-attenuation thyroid nodule, suggesting a fat-containing lesion.

We present a case where cytologic reports of FNA indicated atypia of undetermined significance. Subsequent minimally invasive video-assisted thyroidectomy (MIVAT) confirmed the diagnosis of thyroid lipoadenoma.

## Case presentation

**Figure 1:**
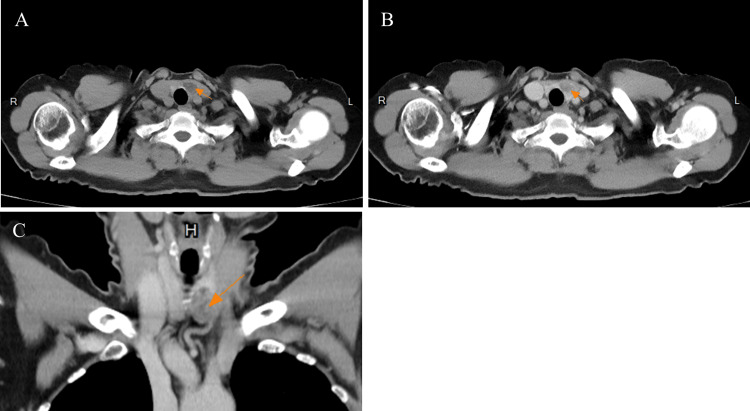
Chest CT with contrast of the left thyroid nodule A-C: The CT revealed a low-attenuation nodule measuring 2.5 x 1.7 x 1.2 cm in the left thyroid gland (orange arrow), with mild enhancement noted after contrast injection.

 A 74-year-old Chinese female patient with a medical history of left breast cancer, type II diabetes mellitus, and hypertension received chest CT during her routine breast cancer surveillance, and a low-attenuation and heterogenous thyroid nodule in the left lobe of the thyorid gland was incidentally noted; this had mild enhancement after contrast injection (Figure 1). The patient was asymptomatic, without any tracheal compression symptoms.

**Figure 2:**
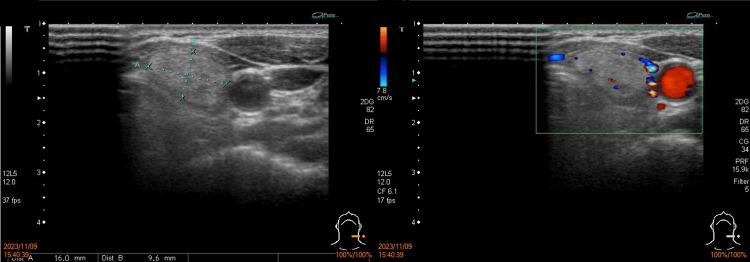
Sonogram of the left thyroid nodule A well-encapsulated and hyper-isoechoic solid lesion with a smooth margin, measuring 16 x 9.6 mm, was detected in the left thyroid gland under sonogram. It was wider than tall and showed hypovascularity under Doppler sonography, and was characterized as EU-TIRADS 2. EU-TIRADS: European Thyroid Imaging Reporting and Data System

Laboratory tests showed euthyroid levels with thyroglobulin at 20.46 ng/mL, free thyroxine at 1.22 ng/dL, triiodothyronine at 94.33 ng/dL, and thyroid-stimulating hormone at 3.64 μIU/mL (Table 1). Sonography revealed a solid, well-encapsulated, homogeneous nodule, measuring 16 x 9.6 mm, with a smooth margin in her left thyroid gland, characterized as European Thyroid Imaging Reporting and Data System (EU-TIRADS) 2 (Figure 2). Subsequent ultrasound-guided FNA yielded a cytologic report indicating atypia of undetermined significance. Given the potential risk of malignancy, she received MIVAT of the left thyroid.

**Table 1:** The preoperative thyroid function test results

Test	Result	Normal range
Thyroglobulin	20.46 ng/mL	< 50 ng/mL
Free thyroxine (free T4)	1.22 ng/dL	0.89 ~ 1.79 ng/dL
Triiodothyronine (T3)	94.33 ng/dL	78 ~ 182 ng/dL
Thyroid-stimulating hormone (TSH)	3.64 3.64 μIU/mL	0.17 ~ 4.05 μIU/mL

**Figure 3:**
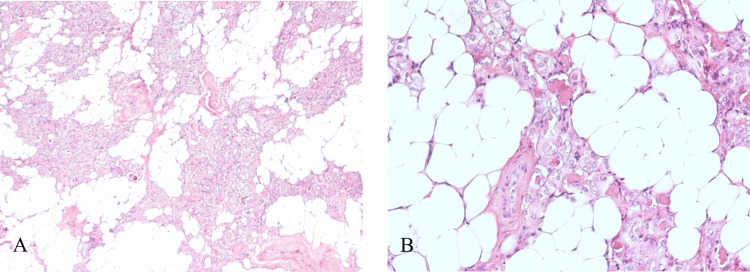
The pathology slides of the thyroid lipoadenoma A-B: The thyroid lipoadenoma under H&E stain revealed that the tumor was mainly composed of adipose tissue and follicular cells, with an adipose tissue volume of 40% to 50%. H&E: Hematoxylin and eosin

The specimen of the left thyroid measured 3.7 x 2.0 x 1.0 cm and weighed 4.7 grams. Grossly, the thyroid was enlarged and well-circumscribed. Microscopic examination revealed a well-circumscribed nodule with abundant adipose tissue and many colloid-rich follicles lined by flattened to cuboidal epithelium, consistent with the diagnosis of thyroid lipoadenoma (Figure 3). The volume of the adipose tissue was around 40% to 50% of the thyroid lipoadenoma. After MIVAT for one month, the patient maintained normal thyroid function without any complications.

## Discussion

The thyroid is a fat-scarce organ, though intrinsic adipose tissue can be occasionally found in subcapsular and perivascular areas. Fat-containing lesions in the thyroid are rare, with an incidence rate estimated between 0.98% and 2.8% [4]. These lesions can be related to non-neoplastic conditions, such as nodular hyperplasia, amyloid goiter, Grave’s disease, or lymphocytic thyroiditis, as well as neoplastic lesions, including thyroid lipoadenoma, papillary carcinoma, and liposarcoma. Hence, thyroid lipoadenoma is a further uncommon neoplasm in the thyroid gland, composed of adipose tissue and thyroid tissue. A study states that thyroid lipoadenomas are more prevalent in women in their fifties. The nodules are more likely to be found in the right lobe than the left lobe; nodules at the isthmic region are relatively rare. The median size of the tumor was 2.75 cm, and the volume of adipose tissue ranged from 10% to 90% [5].

In most cases, the diagnosis of a thyroid lesion is confirmed through clinical symptoms, laboratory data, sonographic findings, and FNA cytologic reports. Patients with thyroid lipoadenoma typically remain asymptomatic and euthyroid, while others experience cervical enlargement and compression symptoms, including dyspnea and dysphagia [1], which may exhibit tracheal deviation and airway obstruction [2].

The adipose tissue may appear isoechoic in a sonogram, making it difficult to differentiate from normal thyroid parenchyma [6]. The thyroid lipoadenoma in CT presents as a low-attenuation lesion, which may suggest lipomatosis and support the diagnosis. Fine-needle aspiration is an effective procedure for evaluating a thyroid mass. In patients with thyroid lipoadenoma, the cytologic report may reveal a mixture of adipocytes and follicular cells. Nonetheless, most of these lesions are not recognized as fat-containing lesions in the thyroid for several reasons. First, low cytologic material in FNA specimens leads to low accuracy, complicating the diagnosis. Second, the adipose tissue may be mistakenly thought to originate from perithyroid tissue or subcutaneous tissue due to its rarity. Third, searching for adipocytes in an aspiration specimen is not a routine task, and a relative lack of awareness of thyroid lipoadenoma may cause the adipose tissue to be overlooked [3]. These factors increase the difficulty of preoperative diagnosis of thyroid lipoadenoma. For patients with suspected thyroid lipoadenoma, common diagnostic tools for thyroid nodules, such as sonogram and FNA, provide limited assistance in diagnosing thyroid lipoadenoma, necessitating thyroidectomy for definite diagnosis and treatment.

## Conclusions

Thyroid lipoadenoma is a rare, benign, fat-containing neoplasm of the thyroid gland. It is well-circumscribed and often asymptomatic, though some patients present with compression symptoms. Both sonography and FNA are limited in confirming the diagnosis of thyroid lipoadenoma. A CT may be helpful if a well-defined, low-attenuation lesion is detected, supporting the presence of intrathyroidal fat. However, a pathological examination is still necessary for a definitive diagnosis. As a result, thyroidectomy may be essential for both confirming the diagnosis and providing definitive treatment for patients with thyroid lipoadenoma.
